# Hypoxia Promotes Vascular Smooth Muscle Cell (VSMC) Differentiation of Adipose-Derived Stem Cell (ADSC) by Regulating Mettl3 and Paracrine Factors

**DOI:** 10.1155/2020/2830565

**Published:** 2020-02-20

**Authors:** Jiaying Lin, Qianqian Zhu, Jialyu Huang, Renfei Cai, Yanping Kuang

**Affiliations:** Department of Assisted Reproduction, Shanghai Ninth People's Hospital, Shanghai Jiao Tong University School of Medicine, Shanghai 200011, China

## Abstract

Adipose-derived stem cell (ADSC) is an alternative and less invasive source of mesenchymal stem cells which can be used to develop biological treatment strategies for tissue regeneration, and their therapeutic applications hinge on an understanding of their physiological characteristics. N6-Methyladenosine (m6A) is the most common chemical modification of mRNAs and has recently been revealed to play important roles in cell lineage differentiation and development. However, the role of m6A modification in the vascular smooth muscle cell (VSMC) differentiation of ADSCs remains unclear. Herein, we investigated the expression of N6-adenosine methyltransferases (Mettl3) and demethylases (Fto and Alkbh5) and found that Mettl3 was upregulated in ADSCs undergoing vascular smooth muscle differentiation induction. Moreover, silence of Mettle3 reduced the expression level of VSMC-specific markers, including *α*-SMA, SM22*α*, calponin, and SM-MHC. Meanwhile, Mettl3 knockdown also decreased the expression of paracrine factors, including VEGF, HGF, TGF-*β*, GM-CSF, bFGF, and SDF-1. In addition, our results suggested that hypoxia stress promotes the ADSC differentiate into VMSCs and regulates the secretion of VEGF, HGF, TGF-*β*, GM-CSF, bFGF, and SDF-1 by mediating Mettl3 gene expression. These observations might contribute to novel progress in understanding the role of epitranscriptomic regulation in the VSMC differentiation of ADSCs and provide a promising perspective for new therapeutic strategies for tissue regeneration.

## 1. Introduction

N6-Methyladenosine (m6A), a methylation modification at the N6 position of adenosine in RNAs, is the most abundant modification in eukaryotic mRNAs [1, 2]. As a dynamic reversible process in mammals, methyltransferases METTL3 and METTL14, two m6A “writers,” form a complex to mediate the addition of methyl groups to adenosines in target RNAs [3], whereas the demethylation is mediated by two “erasers”: FTO and ALKBH5 [4, 5]. Additionally, YTH domain family proteins are typical m6A “readers” proteins, which were responsible for recognizing the m6A-modified transcripts to regulate the downstream effect of the m6A modification [6]. Previous studies have demonstrated that m6A modification plays a crucial role in mRNA metabolism and function, including mRNA stability [7, 8], localization [9], and translation [10, 11]. Growing evidence has confirmed that m6A methylation affects a variety of biological processes, such as proliferation and differentiation of embryonic [12, 13], stress response [14], and learning [15].

Mesenchymal stem cells (MSCs) are a heterogeneous population of nonhematopoietic adult stem cells that present in a verity of tissues, such as bone marrow, adipose, and muscle [16, 17]. They have the property of self-renewal and multilineage differentiation capacity and have been widely utilized in stem cell transplantation, gene therapy, tissue engineering, and immunotherapy [18–20]. Human adipose-derived stem cells (ADSCs) are a type of MSCs isolated from adipose tissue, which demonstrate the multidirectional differentiation potential and have multiple advantages of abundant storage *in vivo*, easy acquisition, and expansion [18–20], suggesting that a broader source of stem cells is available for application in tissue engineering. Furthermore, many studies have demonstrated ADSCs have the potential to induce differentiation into smooth muscle cells [21, 22].

Adipose stem cells, as cells with multidirectional differentiation ability, can differentiate into smooth muscle cells under certain conditions. Mizuno found that ADSCs express smooth muscle cell-specific genes in the myogenic environment [23]. Lien et al. found that MSCs differentiate into smooth muscle cell subtypes under the induction of TGF-*β*1 [24]. It has been reported that TGF-*β* regulates smooth muscle differentiation by directly binding to its type I receptor and activating its downstream Smad signal [24]. Hayashi et al. reported that TGF-*β*1 maintains smooth muscle cell subtype differentiation through the phosphatidylinositol 3-kinase signaling pathway [25]. In addition, bone morphogenetic protein-4 (BMP4) may initiate differentiation of ADSCs into smooth muscle cells indirectly, independent of the TGF-*β* pathway. Lagna et al. reported that BMP4 plays a major role in the transformation of smooth muscle cells from synthetic to contractile cells, and only complete RhoA/ROCK signaling can complete this process, while inhibitors of the TGF-*β* pathway are unable to block this signal [26]. Moreover, recent study revealed that LncRNA HULC promotes smooth-muscle-like differentiation of ADSCs by elevation of BMP9 [21].

However, little is known about the role of m6A modification in the smooth muscle cell differentiation of ADSCs. The connection between hypoxia affects the VSMC differentiation process of ADSCs, and m6A modification also remains to be determined. Therefore, the aims of this study were to explore the effect of m6A methylation on the VSMC differentiation of ADSCs and its role in response to hypoxia.

## 2. Material and Methods

### 2.1. Isolation, Culture, and Characterization of ADSCs

Fresh human subcutaneous adipose tissue was obtained from patients who underwent abdominal liposuction in accordance with procedures approved by the ethics committee, with an average age of 25 years. The adipose tissue was washed three times with sterile phosphate-buffered saline (PBS) buffer and dispensed into a 50 ml centrifuge tube in a volume of 25 ml. Then, the tissue was digested with 0.075% collagenase type I (Gibco, Carlsbad, CA, USA) at 37°C for 60 min on a shaker and centrifuged at 1500 rpm for 10 min. The tissue cells at the bottom of the tube were then resuspended in LG-DMEM growth medium containing 10% fetal bovine serum. Undigested tissue was removed with a 200 mesh screen. The cells were seeded at a density of 4 × 10^4^/cm^2^ on a cell culture dish of 100 mm in diameter and cultured at 100% humidity, 5% CO^2^, and 37°C. After 24 hours, the original culture solution was aspirated, and PBS was washed twice to remove blood cells and unattached cells. Then, change the liquid every other day. The cells were passaged when ~80~90% confluence was reached, and those cells at passage 3 were used in the following study. The expression levels of different cell surface markers, including CD13+, CD44+, CD90+, CD45-, and CD34-, were determined by flow cytometry. The osteogenic and adipogenic differentiations of ADSCs were conducted to determine the multilineage differentiation of ADSCs by Alizarin Red and Oil Red O staining, respectively.

### 2.2. Induction of VSMC Differentiation

Adipose-derived stem cells at passage 3 were seeded in 60 mm culture dishes, and when grown to 60% confluence, the growth medium was discarded and washed three times with PBS. The cells were cultured for 24 hours by adding starvation solution (1% FBS in low-sugar DMEM medium) one day before cell induction. Then, smooth muscle cell-inducing solution (LG-DMEM, 1% FBS, 5 ng/ml of TGF-*β*1, and 2.5 ng/ml of BMP4) was added for 7 days, and the solution was changed every other day. The growth and morphological changes of the cells were observed under a microscope every day.

### 2.3. Osteogenic and Adipogenic Differentiation of ADSCs

For osteogenic differentiation, 5 × 10^3^ cells/cm^2^ were seeded in a T75 flask, replacing the growth medium after 2 days with Complete STEMPRO Osteogenesis Differentiation Medium (Gibco). After 14 and 21 days, cells were stained with Oil Red O to stain lipids. ADSCs were differentiated toward the adipogenic lineage by seeding 1 × 10^4^ cells/cm^2^ in a T75 flask and replacing the culture medium after 2 days with Complete Adipogenesis Differentiation Medium (Gibco). After 14 and 21 days, cells were processed for Alizarin Red S staining in order to detect the calcium deposits in the culture.

### 2.4. Mettl3 Knockdown Using shRNA Transfection

Transfection of plasmids was carried out using Lipofectamine 2000 (Invitrogen) according to the manufacturer's instructions. The method of Mettl3 knockdown reported in previous article was used in this study [27]. Briefly, siRNA target sequence of Mettl3, AGTCACAAACCAGATGAAATA, and a nonspecific shRNA construct were designed and cloned into a hU6-MCS-Ubiquitin-EGFP-IRES-puromycin vector, and the vectors were then transfected into 293FT cells. Viruses were collected at 48 h after transfections and then transduced into ADSCs. Cells were incubated in solution with puromycin (1 *μ*g/ml, Sigma) for 2 weeks, and stable clones were maintained in 0.5 *μ*g/m puromycin to ensure a >90% transfection rate. Western blotting assay was performed to confirm the silence effect of Mettl3. The cells were independently divided into control (untransduced group), shCtrl (negative control group), and shMettl group. In addition, the cells of the shCtrl and shMettl were cultured under 21% (normoxia) or 1% (hypoxia) O^2^ conditions.

### 2.5. Western Blot Analysis

Homogenization was performed using lysis buffer (50 mM Tris (pH 7.4), 150 mM NaCl, 1% TritonX-100, 1% sodium deoxycholate, 0.1% SDS, and 2 mM sodium pyrophosphate). Proteins were then loaded for electrophoresis, and those proteins on gel that were transferred to an PVDF membrane were blocked by 10% skimmed-milk for 30 min. Proteins on the membrane were incubated with anti-Mettl3 (1 : 1000), anti-Fto (1 : 1000), anti-Alkbh5 (1 : 1000), anti-SM22a (1: 1000), anti-*α*-SMA (1: 1000), anti-calponin (1: 1000), and anti-SM-MHC (1: 1000), and anti-GAPDH antibodies (CST, MA, USA) overnight at 4°C and aspirated primary antibody and rinsed with TBST for 5 minutes, then incubated with secondary antibodies for 2 h. Analysis of protein expression was carried out with bands on membranes using ECL reagent (Pierce, IL, USA). The data are from 3 independent experiments.

### 2.6. Alizarin Red S Staining

ADSCs seeded in 6-well plates were induced in osteogenic medium for 14 days to detect mineralized nodule formation, and the cells were rinsed with PBS and fixed in 4% paraformaldehyde solution for 20 min after culturing. Then, the cells were stained with 1% Alizarin Red S solution at room temperature for 10 min and washed 5 times. Mineralized nodules were photographed under a microscope (Axiovert 40C; Carl Zeiss Inc., Jena, Germany).

### 2.7. Oil Red O Staining

After 14 days of in vitro osteogenic induction, cells were washed twice with PBS and subsequently fixed with 4% neutral formaldehyde for 30 min at room temperature. Fixed cells were then stained with Oil Red O (Sigma-Aldrich) at room temperature. Cells were washed again in PBS and observed under a light microscope (magnification, ×200).

### 2.8. Quantitative Real-Time PCR (qPCR)

The total RNA from ADSCs was extracted using TRIzol reagent (Invitrogen, Carlsbad, NM, USA), and the cDNA was synthesized using the SuperScript IV First Strand Synthesis System (Invitrogen, Carlsbad, NM, USA) and used in the following determination of gene expression. PCR was carried out and monitored using the LightCycler 480, and the primers used in this study are listed in Table 1.

### 2.9. ELISA

The quantitation of VEGF, HGF, TGF-*β*, GM-CSF, bFGF, and SDF-1 in the conditioned medium was performed by using a VEGF Human ELISA Kit (Invitrogen), HGF Human ELISA Kit (Invitrogen), TGF-*β* ELISA Kit (Gaithersburg, MD), GM-CSF ELISA Kit (Medical Resources Ltd., Surrey Hills, NSW, Australia), bFGF ELISA Kit (Amersham, Buckinghamshire, UK), and SDF-1Quantikine kit (R&D), respectively.

### 2.10. m6A Detection

N6-Methyladenosine levels of cells were quantified using the EpiQuik m6A RNA Methylation Quantification Kit (EpiQuik, USA) according to the manufacturer's instruction. Briefly, total RNA is bound to wells using the RNA high-binding solution. m6A is detected using capture and detection antibodies. The m6A level was then quantified by reading the 450 nm absorbance.

### 2.11. Statistical Analyses

All experiments were conducted in triplicate. All data are presented as the mean ± standard deviation (SD) and were analyzed using the Stata SE15.0 (Stata Corporation). One-way analysis of variance was used to compare the experimental groups. *p* value < 0.05 was considered as statistical significance.

## 3. Results

### 3.1. Selection and Identification of ADSCs

**Figure 1:**
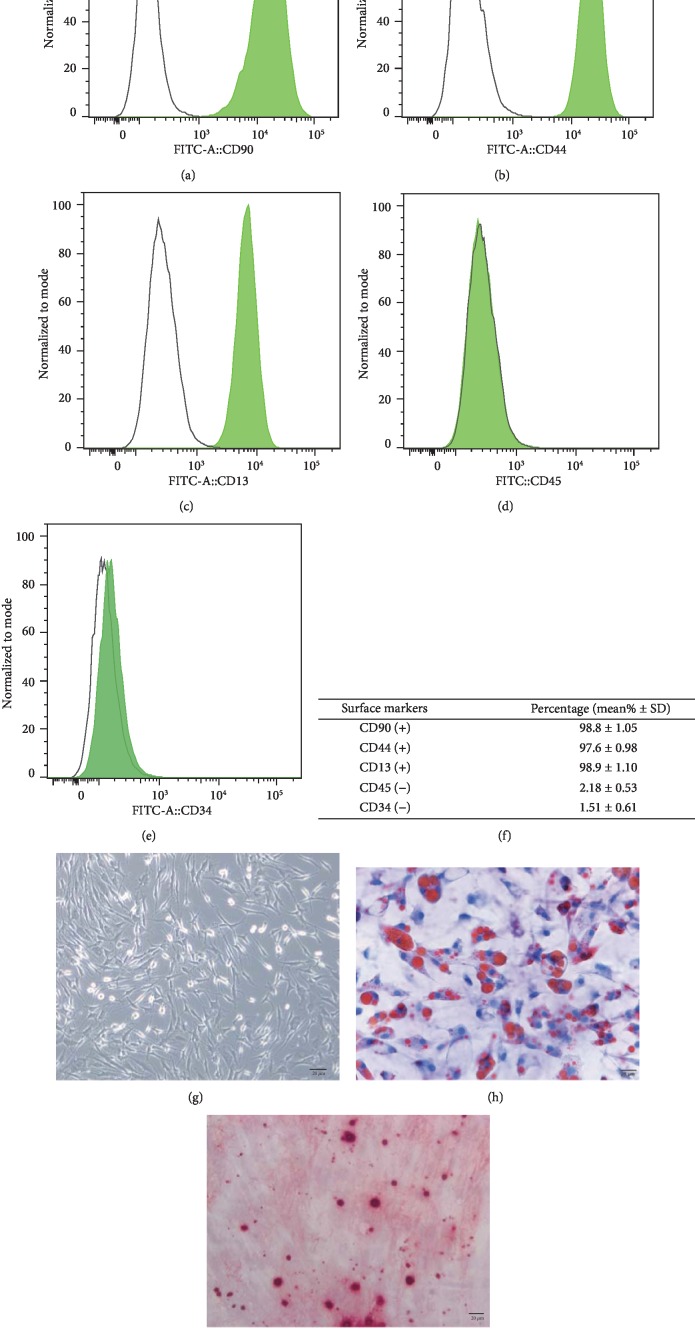
Characterization of ADSCs. (a–e) The expression of cell surface markers were evaluated by flow cytometry. Average of cells (%) is shown in (f) as mean% ± S.D. (standard deviation). (g–i) The differentiation ability of ADSCs towards osteogenesis-like or adipogenesis-like cells was assessed by Alizarin Red staining (h) or Oil Red O staining (i). The “black” scale bars represent 20 *μ*m (original magnification ×200).

Human adipose-derived stem cells were successfully isolated, and the morphology of ADSCs was identified, as shown in Figure 1. The results showed a positive expression of CD13, CD90, and CD44, but negative for CD34, and CD45, indicating that the ADSCs were of high purity and excluding the contamination of endothelial and hematopoietic (Figures 1(a) and 1(b)). Moreover, the ADSCs were able to be differentiated into osteoblast-like cells and adipose-like cells when cultured in a differentiating induction medium, as determined by Alizarin Red staining and Oil Red O staining, respectively (Figures 1(h) and 1(i)). These data confirmed the status of the isolated and cultured ADSCs.

### 3.2. Expression of m6A Methyltransferase and Demethylases in ADSCs Undergoing VSMC Differentiation

**Figure 2:**
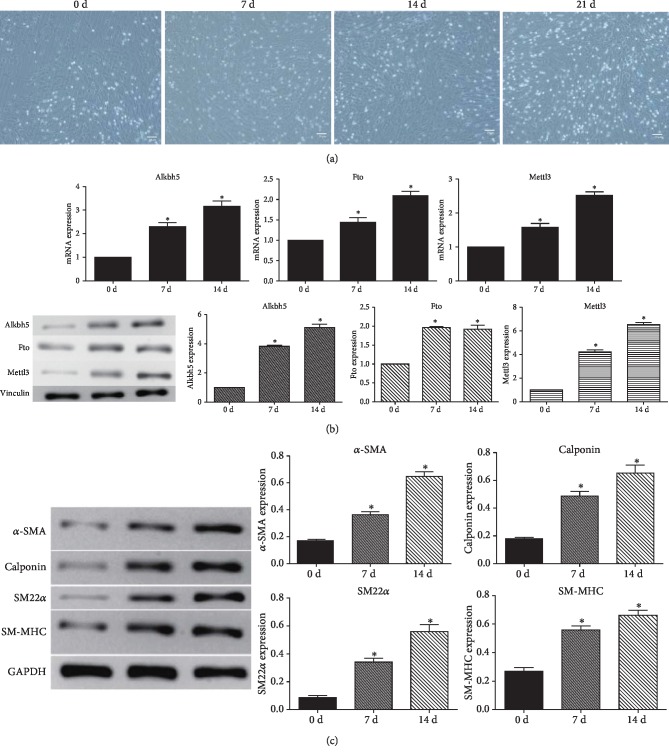
Differentiation of ADSCs and expression of m6A methyltransferase and demethylases. (a) Photomicrographs of the VSMC differentiation from ADSCs at 0, 7, 14, and 21 days. (b) The expression level of mRNA and protein of Alkbh5, Fto, and Mettl3 in ADSCs after 7 and 14 days of induction. (c) The expression of a protein level of 4 VSMC-specific markers after 7 and 14 days of induction.

During the VSMC differentiation of ADSCs, cells were cultured in an induction medium at the indicated time points, and the cell morphology was observed under a microscope. The third to fifth generation of adipose stem cells appeared as fibroblast-like spindle cells under the microscope. After induction by TGF-*β*1+BMP4, the cells were still spindle-shaped, but slightly shorter and flatter than adipose-derived stem cells, showing “peak-valley”-like growth in the microscope field (Figure 2(a)).

To reveal the role of m6A modification in the differentiation potential of ADSCs, the expression pattern and m6A methyltransferase (Mettle3) and demethylases (Fto, Alkbh5) were measured during the induction of ADSCs. Both the mRNA and protein levels of Mettl3 and Alkbh5 elevated after 7 and 14 days of induction (Figure 2(b)). Although Fto increased at the mRNA level after 7 and 14 days, its protein level after 14 days of induction showed no significant difference when compared with the protein level after 7 days of induction. Accordingly, the expression pattern of Mettl3 and Alkbh5 during differentiation was consistent with those of the mineralization-related markers (Figure 2(c)).

### 3.3. Effect of Mettl3 Knockdown on the VSMC Differentiation Potential of ADSCs

**Figure 3:**
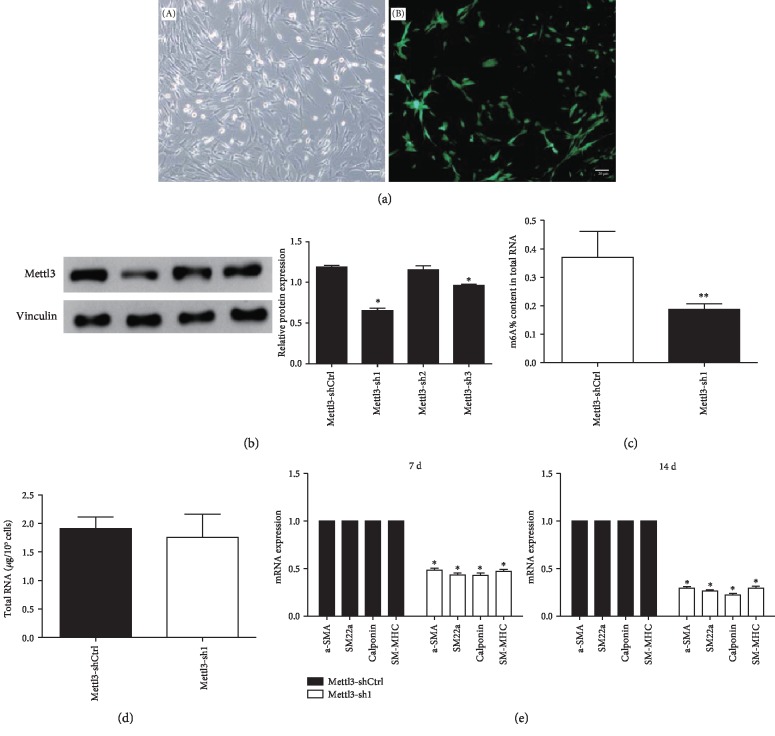
Effect of Mettl3 knockdown on the VSMC differentiation potential of ADSCs. (a) Fluorescence protein marker was used to determine the transfer efficiency of Mettl3 knockdown in ADSCs. After transfection for 72 h, the cells were observed under a microscope (A). (B) is an immunofluorescence image taken at the same time. The “white” scale bars represent 20 *μ*m (original magnification ×200). (b) The expression level of Mettl3 was determined using Western blotting in the Mettl3-shRNA and Mettl3-shCtrl groups. Vinculin was used as an internal control. The band intensities were analyzed using the ImageJ software. (c) The m6A level of ADSCs in the Mettle3-shaCtrl and Mettl3-sh1 groups. (d) The total RNA level of ADSCs in the Mettle3-shaCtrl and Mettl3-sh1 groups. (e) The mRNA expression level of *α*-SMA, SM22*α*, calponin, and SM-MHC in the Mettl3-shRNA and Mettl3-shCtrl groups was assessed using qRT-PCR after 7 and 14 days of induction. GAPDH was used as an internal control. All of the results represent the mean ± standard deviation of three independent experiments (*n* = 3). Significant difference compared with the control (^∗^*p* < 0.05).

To investigate the role of m6A in the VSMC differentiation process of ADSCs, the Mettl3 was selected to conducted knockdown experiments. Specific shRNAs were applied to knock down the expression of Mettl3 in ADSCs, and quantification of lentiviral gene transfer efficiency in ADSCs was measured via the proportion of fluorocytes. A transfer efficiency up to 90% was achieved at 48 h after transfection (Figure 3(a)). The Mettl3 protein level exhibited an approximate 55% decrease in the shRNA group (#sh1) compared with the negative control group, suggesting that Mettl3 was effectively silenced in ADSCs (Figure 3(b)). In addition, silence of Mettl3 has no significant effect on the levels of total RNA (Figure 3(c)), whereas the m6A level was significantly decreased while Mettl3 was silenced in ADSCs (Figure 3(d)). Mettl3-sh1 was thus chosen for further experiment.

To investigate the differentiation potential of ADSCs after Mettl3 knockdown, the expression level of several VSMC-specific markers was measured. The results showed that Mettl3 knockdown reduced the mRNA level of alpha-smooth muscle actin (*α*-SMA), smooth muscle 22 alpha (SM22*α*), calponin, and cardiac myosin heavy chain (SM-MHC) in ADSCs after differentiation induction for 7 and 14 days (Figure 3(e)). The results of immunofluorescence staining of *α*-SMA, SM22*α*, calponin, and SM-MHC showed the same trend.

### 3.4. Hypoxia Upregulates Mettl3 to Promote the VSMC Differentiation of ADSCs

**Figure 4:**
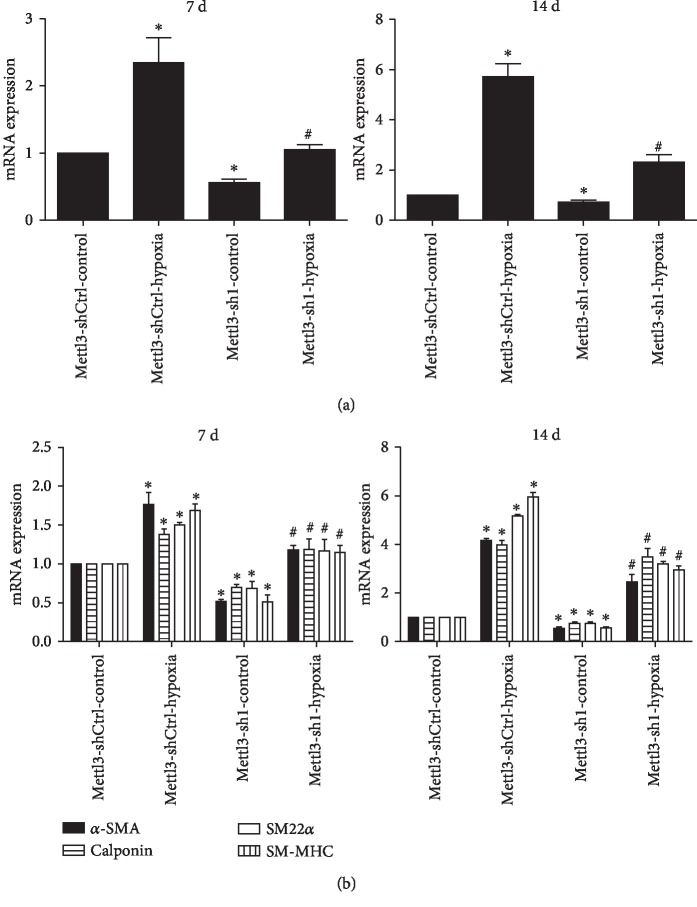
Hypoxia promotes VSMC differentiation form ADSCs by upregulating the expression of Mettl3. (a) Hypoxia promotes the ADSC differentiate into VSMCs. (b) The mRNA and protein levels of Mettl3 were increased under hypoxia condition. (c) The mRNA levels of *α*-SMA, SM22*α*, calponin, and SM-MHC were increased under hypoxia condition. All of the results represent the mean ± standard deviation of three independent experiments (*n* = 3). Significant difference compared with the control (^∗^*p* < 0.05).

To investigate the effect of hypoxia on the VSMC differentiation of ADSCs, the expression level of Mettl3 and several VSMC-specific markers was measured. The results showed that hypoxia condition promoted ADSC differentiate into VSMCs. The mRNA level of *α*-SMA, SM22*α*, calponin, and SM-MHC increased in ADSCs after differentiation induction for 7 and 14 days under hypoxia. Moreover, the mRNA and protein levels of Mettl3 presented the same increased trend after induction under hypoxia (Figure 4(a)). In addition, compared with the Mettl3-sh1 group, hypoxia stress could promote ADSC differentiate into VSMCs, and the mRNA level of *α*-SMA, SM22*α*, calponin, and SM-MHC increased in ADSCs after differentiation induction for 7 and 14 days under hypoxia (Figure 4(b)). Accordingly, the expression pattern of Mettl3 during differentiation was consistent with those of the VSMC-specific markers. The results suggested that hypoxia promoted the VSMC differentiation of ADSCs by mediating Mettl3.

### 3.5. Effect of Hypoxia on the Expression of Paracrine Factors

To explore the influence of hypoxia on the paracrine manner, the expression level of several paracrine factors was measured by enzyme-linked immunosorbent assay (ELISA). The results showed that hypoxia increased the expression level of vascular endothelial growth factor (VEGF), hepatocyte growth factor (HGF), transforming growth factor *β* (TGF-*β*), granulocyte-macrophage colony-stimulating factor (GM-CSF), basic fibroblast growth factor (bFGF), and stromal cell-derived factor-1 (SDF-1) (Table 2). Moreover, knockdown of Mettl3 decreased the expression of VEGF, HGF, TGF-*β*, GM-CSF, bFGF, and SDF-1. To sum up, the results suggested that Mettl3 could reduce the expression of several paracrine factors, whereas hypoxia stress might invert this process to promote the VSMC differentiation from ADSCs.

## 4. Discussion

m6A is a high frequent epitranscriptomic modification in mRNAs and widely occurs in single-cell organisms, plants, and vertebrates [28, 29]. RNA m6A-methylated modification is mediated by a core complex consisting of METTL3, METTL4, and WTAP and is removed by FTO and ALKBH5. Growing evidence has proved that m6A involves in regulating the pluripotency and differentiation of ESCs and somatic cell reprogramming. It was reported that m6A methylation regulates histone modification to participate in the self-renewal process of embryonic neural stem cell [30]. Recent study revealed the indispensable role of m6A during the differentiation of hematopoietic stem cell [31]. Additionally, Mettl3-mediated m6A was proved to regulate spermatogonial differentiation and meiosis initiation [32].

In recent years, the application of ADSCs for reconstructive and plastic surgery has been exceedingly increased. ADSC is an important kind of mesenchymal stem cell (MSC) and could differentiate into lipocytes [33], osteocytes [34], and VSMCs [35]. In the application of tissue engineering, ADSCs were able to differentiate into many kinds of phenotypes to repair damaged tissue. In addition, ADSCs could secrete various proteins, cytokines, and growth factors to stimulate the migration, proliferation, and differentiation of local cells of damaged area. Till now, the underlying molecular mechanism has not yet been elucidated [36]. To explore the role of m6A in the VSMC differentiation of ADSCs, ADSCs were cultured using smooth muscle cell inducing solution to establish a VSMC model. The expression levels of main m6A methyltransferase and demethylases, including Mettl3, Fto, and Alkbh5, were then evaluated. The results showed that the mRNA and protein levels of Mettl3 and Alkbh5 increased after VSMC induction, but the protein expression of Fto and Alkbh5 did not significantly differ with or without VSMC differentiation. The results suggested that Mettl3-dependent RNA methylation might participate in the VSMC differentiation of ADSCs.

METTL3 is the main methyltransferase critical for m6A methylation [37]. Deletion or overexpression of Mettl3 alters the total m6A methylation level, which has a direct effect on cell survival, stem cell maintenance, and lineage determination [12, 38]. Depletion of the Mettl3 homolog in Arabidopsis thaliana gives change to the growth patterns and reduced apical dominance. The knockdown of Drosophila Mettl3 homolog (Dm ime4) has been proven to suppress oogenesis. Mettl3 inhibition results in a concomitant decrease in the cellular m6A level and apoptosis of human Hela cells [39]. To elucidate the role of Mettl3 in the VSMC differentiation of ADSCs, mettl3 was silenced in ADSCs, and the effect of Mettl3 knockdown on cell differentiation was investigated in the present study. The results showed that inhibition of Mettl3 could reduce the expression of VSMC-specific markers such as *α*-SMA, SM22*α*, calponin, and SM-MHC. Moreover, we determined the effect of Mettl3 on the expression of paracrine factors. The results demonstrated a decreased level of VEGF, HGF, TGF-*β*, GM-CSF, bFGF, and SDF-1 after silencing Mettl3 gene expression. Therefore, the above data suggested that Mettl3 increased the VSMC differentiation of ADSCs and affected the expression of paracrine factors which may involve in the cell migration, proliferation, and differentiation.

Accumulating evidence suggested that hypoxia is beneficial for cell survival, proliferation, and migration *in vitro*. Buizer et al. reported that, compared with normoxia condition, 1% or 2% oxygen promoted the proliferation of MSCs and the expression of angiogenic factors [40]. In addition, it was also proved that hypoxia promotes the proliferation of MSC and ADSCs [41, 42]. Furthermore, the scratch and transwell assays were performed by Yu et al., which indicated that 1% oxygen with low-dose inflammatory stimuli can synergistically enhance the migration of BMSC [43]. In the present study, we investigated the effect of hypoxia on the VSMC differentiation from ADSCs and the role of m6A in this process. The results showed that hypoxia promoted the ADSCs to differentiate into VSMCs. Meanwhile, silence of Mettl3 could decrease the effect of hypoxia on the VSMC differentiation of ADSCs, suggesting that hypoxia involved in the differentiation process by mediating the expression of Mettl3. Moreover, hypoxia stress elevated the expression of several paracrine factors, including VEGF, HGF, TGF-*β*, GM-CSF, bFGF, and SDF-1, which would eventually participate in the VEGF, HGF, TGF-*β*, GM-CSF, bFGF, and SDF-1. Further studies are required to elucidate the molecular mechanism in the differentiation process of ADSCs.

## 5. Conclusion

In conclusion, the present work estimated the expression pattern of m6A methyltransferase and demethylases during the VSMC differentiation of ADSCs and demonstrated that Mettl3 is highly expressed in the process of VSMC differentiation. Investigating the role of Mettl3 in regulating cell differentiation showed that the loss of Mettl3 suppressed the VSMC differentiation potential of ADSCs. Moreover, hypoxia stress accelerated the differentiation of ADSC into VSMCs, which may be explained with upregulated expression of Mettl3 and paracrine factors. These discoveries might advance novel progress in the role of the epitranscriptome in VSMC differentiation and provide a promising perspective for the development of innovative therapeutic strategies for vascular network regeneration.

## Figures and Tables

**Table 1:** The primers used to determine the expression of genes by RT-PCR.

Gene	Forward primer	Reverse primer
Mettl3	5′CTTTAGCATCTGGTCTGGGCT3′	5′CCTTCTTGCTCTGCTGTTCCT3′
Alkbh5	5′ACCACCAAACGGAAGTACCAG3′	5′TCATCCTGGCTGAAGAGACG3′
Fto	5′ACTGGTTTTCCGAGAGGCTG3′	5′GTGAGCACGTCTTTGCCTTG3′
*α*SMA	5′GGTGATGGTGGGAATGGG3′	5′GCAGGGTGGGATGCTCTT3′
SM22*α*	5′AACAGCCTGTACCCTGATGG3′	CGGTAGTGCCCATCATTCTT3′
Calponin	5′ATGTCCTCTGCTCACTTCA3′	5′TTTCCGCTCCTGCTTCTCT3′
SM-MHC	5′TGCTTTCGCTCGTCTTCC3′	5′CGGCAACTCGTGTCCAAC3′

**Table 2:** The expression of paracrine factors in different groups.

Gene	Mettl3-shCtrl-Ctrll	Mettl3-shCtrl-hypoxia	Mettl3-sh1-Ctrl	Mettl3-sh1-hypoxia
VEGF	268.32 ± 11.32	563.47 ± 23.77^∗^	201.17 ± 10.05^∗^	254.14 ± 9.75^#^
HGF	32.03 ± 6.20	76.15 ± 3.60^∗^	25.11 ± 5.13^∗^	35.33 ± 5.98^#^
TGF-*β*	519.95 ± 12.31	1390.72 ± 38.31^∗^	470.12 ± 10.13^∗^	551.15 ± 11.77^#^
GM-CSF	137.41 ± 3.97	375.67 ± 18.95^∗^	99.98 ± 2.14^∗^	129.67 ± 2.99^#^
bFGF	54.68 ± 3.45	103.51 ± 5.04^∗^	23.89 ± 2.12^∗^	49.78 ± 3.13^#^
SDF-1	166.02 ± 9.38	413.86 ± 9.47^∗^	132 ± 8.87^∗^	178.32 ± 8.18^#^

## Data Availability

The data used to support the findings of this study are included within the article.

## References

[1] Amort T., Rieder D., Wille A. (2017). Distinct 5-methylcytosine profiles in poly(A) RNA from mouse embryonic stem cells and brain. *Genome Biology*.

[2] Zhou Y., Zeng P., Li Y. H., Zhang Z., Cui Q. (2016). SRAMP: prediction of mammalian N6-methyladenosine (m6A) sites based on sequence-derived features. *Nucleic Acids Research*.

[3] Wang P., Doxtader K. A., Nam Y. (2016). Structural basis for cooperative function of Mettl3 and Mettl14 methyltransferases. *Molecular Cell*.

[4] Zhang C., Samanta D., Lu H. (2016). Hypoxia induces the breast cancer stem cell phenotype by HIF-dependent and ALKBH5-mediated m6A-demethylation of NANOG mRNA. *Proceedings of the National Academy of Sciences*.

[5] Jia G., Fu Y., He C. (2013). Reversible RNA adenosine methylation in biological regulation. *Trends in Genetics*.

[6] Xu C., Wang X., Liu K. (2014). Structural basis for selective binding of m6A RNA by the YTHDC1 YTH domain. *Nature Chemical Biology*.

[7] Du H., Zhao Y., He J. (2016). YTHDF2 destabilizes m^6^A-containing RNA through direct recruitment of the CCR4-NOT deadenylase complex. *Nature Communications*.

[8] Ke S., Pandya-Jones A., Saito Y. (2017). m^6^A mRNA modifications are deposited in nascent pre-mRNA and are not required for splicing but do specify cytoplasmic turnover. *Genes & Development*.

[9] Roundtree I. A., Luo G. Z., Zhang Z. (2017). YTHDC1 mediates nuclear export of N6-methyladenosine methylated mRNAs. *eLife*.

[10] Meyer K., Saletore Y., Zumbo P., Elemento O., Mason C., Jaffrey S. (2012). Comprehensive analysis of mRNA methylation reveals enrichment in 3′ UTRs and near stop codons. *Cell*.

[11] Wang X., Zhao B. S., Roundtree I. A. (2015). N(6)-methyladenosine modulates messenger RNA translation efficiency. *Cell*.

[12] Batista P. J., Molinie B., Wang J. (2014). m^6^A RNA Modification Controls Cell Fate Transition in Mammalian Embryonic Stem Cells. *Cell Stem Cell*.

[13] Geula S., Moshitch-Moshkovitz S., Dominissini D. (2015). m6A mRNA methylation facilitates resolution of naïve pluripotency toward differentiation. *Science*.

[14] Engel M., Eggert C., Kaplick P. M. (2018). The role of m^6^A/m-RNA methylation in stress response regulation. *Neuron*.

[15] Koranda J. L., Dore L., Shi H. (2018). Mettl14 is essential for epitranscriptomic regulation of striatal function and learning. *Neuron*.

[16] Teixeira F. G., Carvalho M. M., Sousa N., Salgado A. J. (2013). Mesenchymal stem cells secretome: a new paradigm for central nervous system regeneration?. *Cellular and Molecular Life Sciences*.

[17] Al-Rifai R., Nguyen P., Bouland N. (2019). In vivo efficacy of endothelial growth medium stimulated mesenchymal stem cells derived from patients with critical limb ischemia. *Journal of Translational Medicine*.

[18] Dzobo K., Vogelsang M., Thomford N. E. (2016). Wharton’s Jelly-Derived Mesenchymal Stromal Cells and Fibroblast-Derived Extracellular Matrix Synergistically Activate Apoptosis in a p21-Dependent Mechanism in WHCO1 and MDA MB 231 Cancer Cells *In Vitro*. *Stem Cells International*.

[19] van Zoelen E. J., Duarte I., Hendriks J. M., van der Woning S. P. (2016). TGF*β*-induced switch from adipogenic to osteogenic differentiation of human mesenchymal stem cells: identification of drug targets for prevention of fat cell differentiation. *Stem Cell Research & Therapy*.

[20] Dzobo K., Turnley T., Wishart A. (2016). Fibroblast-derived extracellular matrix induces chondrogenic differentiation in human adipose-derived mesenchymal stromal/stem cells in vitro. *International Journal of Molecular Sciences*.

[21] Li Y., Shan Z., Yang B. (2018). LncRNA HULC promotes epithelial and smooth-muscle-like differentiation of adipose-derived stem cells by upregulation of BMP9. *Pharmazie*.

[22] Li Y., Shan Z., Yang B. (2017). Cathelicidin LL37 promotes epithelial and smooth-muscle-like differentiation of adipose-derived stem cells through the Wnt/*β*-catenin and NF-*κ*B pathways. *Biochemistry (Mosc)*.

[23] Mizuno H. (2010). The potential for treatment of skeletal muscle disorders with adipose-derived stem cells. *Current Stem Cell Research & Therapy*.

[24] Lien S. C., Usami S., Chien S., Chiu J. J. (2006). Phosphatidylinositol 3-kinase/Akt pathway is involved in transforming growth factor-*β*1-induced phenotypic modulation of 10T1/2 cells to smooth muscle cells. *Cellular Signalling*.

[25] Hayashi K. I., Takahashi M., Nishida W. (2001). Phenotypic modulation of vascular smooth muscle cells induced by unsaturated lysophosphatidic acids. *Circulation Research*.

[26] Lagna G., Ku M. M., Nguyen P. H., Neuman N. A., Davis B. N., Hata A. (2007). Control of phenotypic plasticity of smooth muscle cells by bone morphogenetic protein signaling through the myocardin-related transcription factors. *The Journal of Biological Chemistry*.

[27] Tian C., Huang Y., Li Q., Feng Z., Xu Q. (2019). Mettl3 regulates osteogenic differentiation and alternative splicing of Vegfa in bone marrow mesenchymal stem cells. *International Journal of Molecular Sciences*.

[28] Linder B., Grozhik A. V., Olarerin-George A. O., Meydan C., Mason C. E., Jaffrey S. R. (2015). Single-nucleotide-resolution mapping of m6A and m6Am throughout the transcriptome. *Nature Methods*.

[29] Martínez-Pérez M., Aparicio F., López-Gresa M. P., Bellés J. M., Sánchez-Navarro J. A., Pallás V. (2017). Arabidopsism6A demethylase activity modulates viral infection of a plant virus and the m6A abundance in its genomic RNAs. *Proceedings of the National Academy of Sciences*.

[30] Wang Y., Li Y., Yue M. (2018). N^6^-methyladenosine RNA modification regulates embryonic neural stem cell self-renewal through histone modifications. *Nature Neuroscience*.

[31] Lee H., Bao S., Qian Y. (2019). Stage-specific requirement for Mettl3-dependent m^6^A mRNA methylation during haematopoietic stem cell differentiation. *Nature Cell Biology*.

[32] Xu K., Yang Y., Feng G. H. (2017). Mettl3-mediated m^6^A regulates spermatogonial differentiation and meiosis initiation. *Cell Research*.

[33] Wu X., Wang Q., Kang N. (2017). The effects of different vascular carrier patterns on the angiogenesis and osteogenesis of BMSC–TCP-based tissue-engineered bone in beagle dogs. *Journal of Tissue Engineering and Regenerative Medicine*.

[34] Wystrychowski W., Patlolla B., Zhuge Y., Neofytou E., Robbins R. C., Beygui R. E. (2016). Multipotency and cardiomyogenic potential of human adipose-derived stem cells from epicardium, pericardium, and omentum. *Stem Cell Research & Therapy*.

[35] Chen R., Dean D. (2017). Mechanical properties of stem cells from different sources during vascular smooth muscle cell differentiation. *Molecular & cellular biomechanics: MCB*.

[36] Kalinina N., Kharlampieva D., Loguinova M. (2015). Characterization of secretomes provides evidence for adipose-derived mesenchymal stromal cells subtypes. *Stem Cell Research & Therapy*.

[37] Wang C.-X., Cui G.-S., Liu X. (2018). METTL3-mediated m6A modification is required for cerebellar development. *PLOS Biology*.

[38] Ping X. L., Sun B. F., Wang L. (2014). Mammalian WTAP is a regulatory subunit of the RNA N6-methyladenosine methyltransferase. *Cell Research*.

[39] Yue Y., Liu J., He C. (2015). RNA N6-methyladenosine methylation in post-transcriptional gene expression regulation. *Genes & Development*.

[40] Buizer A. T., Bulstra S. K., Veldhuizen A. G., Kuijer R. (2018). The balance between proliferation and transcription of angiogenic factors of mesenchymal stem cells in hypoxia. *Connective Tissue Research*.

[41] Fotia C., Massa A., Boriani F., Baldini N., Granchi D. (2015). Hypoxia enhances proliferation and stemness of human adipose-derived mesenchymal stem cells. *Cytotechnology*.

[42] Burian E., Probst F., Palla B. (2017). Effect of hypoxia on the proliferation of porcine bone marrow-derived mesenchymal stem cells and adipose-derived mesenchymal stem cells in 2- and 3-dimensional culture. *Journal of Cranio-Maxillo-Facial Surgery*.

[43] Yu Y., Yin Y., Wu R. X., He X. T., Zhang X. Y., Chen F. M. (2017). Hypoxia and low-dose inflammatory stimulus synergistically enhance bone marrow mesenchymal stem cell migration. *Cell Proliferation*.

